# 2-(4-Meth­oxy­phen­oxy)acetohydrazide

**DOI:** 10.1107/S160053681202435X

**Published:** 2012-05-31

**Authors:** Gang Liu, Jie Gao

**Affiliations:** a College of Food Engineering, Jilin Teachers’ Institute of Engineering and Technology, 130052 Changchun, Jilin, People’s Republic of China

## Abstract

The title compound, C_9_H_12_N_2_O_3_, was synthesized by the reaction of ethyl 2-(4-meth­oxy­phen­oxy)acetate with hydrazine hydrate in ethanol. In the acetohydrazide group, the N—N bond is relatively short [1.413 (2) Å], suggesting some degree of electronic delocalization in the mol­ecule. In the crystal, mol­ecules are linked into sheets lying parallel to the *ab* plane by N—H⋯N and N—H⋯O hydrogen bonds.

## Related literature

For general background to and the biological activity of hydrazides, see: Khattab (2005 ▶); Ozdemir *et al.* (2009 ▶); Ashiq *et al.* (2009 ▶); Zhang & Shi (2009 ▶). For related structures, see: Dutkiewicz *et al.* (2009 ▶); Fun *et al.* (2009 ▶, 2010*a* ▶,*b* ▶, 2011 ▶).
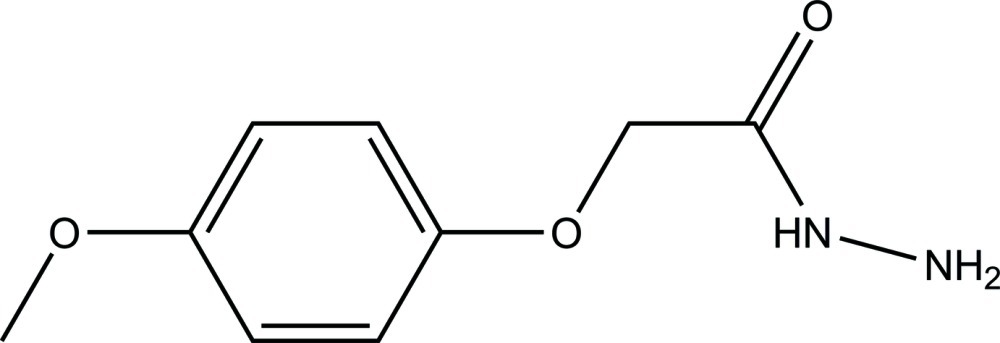


## Experimental

### 

#### Crystal data

C_9_H_12_N_2_O_3_
*M* *_r_* = 196.21
Orthorhombic, 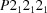

*a* = 4.0964 (17) Å
*b* = 6.382 (3) Å
*c* = 35.608 (14) Å
*V* = 930.9 (7) Å^3^
*Z* = 4
Mo *K*α radiation
μ = 0.11 mm^−1^
*T* = 298 K
0.30 × 0.25 × 0.18 mm

#### Data collection

Bruker SMART APEXII CCD diffractometer
Absorption correction: multi-scan (*SADABS*; Bruker, 2005 ▶) *T*_min_ = 0.969, *T*_max_ = 0.981
4782 measured reflections
1631 independent reflections
1503 reflections with *I* > 2σ(*I*)
*R*_int_ = 0.021

#### Refinement

*R*[*F* ^2^ > 2σ(*F* ^2^)] = 0.030
*wR*(*F* ^2^) = 0.089
*S* = 0.88
1631 reflections
140 parameters
H atoms treated by a mixture of independent and constrained refinement
Δρ_max_ = 0.09 e Å^−3^
Δρ_min_ = −0.15 e Å^−3^

### 

Data collection: *APEX2* (Bruker, 2005 ▶); cell refinement: *SAINT* (Bruker, 2005 ▶); data reduction: *SAINT*; program(s) used to solve structure: *SHELXTL* (Sheldrick, 2008 ▶); program(s) used to refine structure: *SHELXTL*; molecular graphics: *SHELXTL*; software used to prepare material for publication: *SHELXTL*.

## Supplementary Material

Crystal structure: contains datablock(s) global, I. DOI: 10.1107/S160053681202435X/rk2352sup1.cif

Structure factors: contains datablock(s) I. DOI: 10.1107/S160053681202435X/rk2352Isup2.hkl

Supplementary material file. DOI: 10.1107/S160053681202435X/rk2352Isup3.cml

Additional supplementary materials:  crystallographic information; 3D view; checkCIF report

## Figures and Tables

**Table 1:** Hydrogen-bond geometry (Å, °)

*D*—H⋯*A*	*D*—H	H⋯*A*	*D*⋯*A*	*D*—H⋯*A*
N2—H2*A*⋯O3^i^	0.89 (2)	2.51 (2)	3.155 (2)	130.4 (16)
N1—H1⋯N2^ii^	0.88 (2)	2.18 (2)	2.984 (2)	152.2 (18)
N2—H2*B*⋯O3^iii^	0.91 (2)	2.13 (2)	3.027 (2)	167.5 (18)

Symmetry codes: (i) 
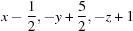
; (ii) 
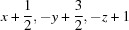
; (iii) 
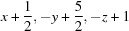
.

**Fig. 1.**
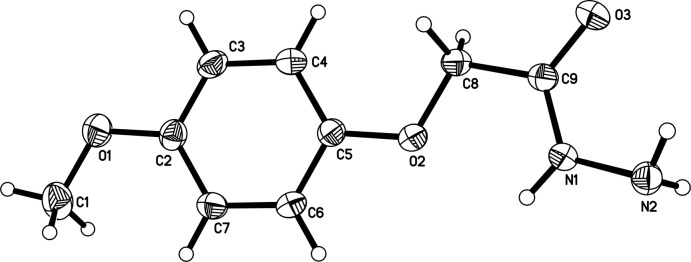
The molecular structure of title compound with the atom numbering scheme. Displacement ellipsoids are drawn at the 30% probability level. H atoms are presented as a small spheres of arbitrary radius.

**Fig. 2.**
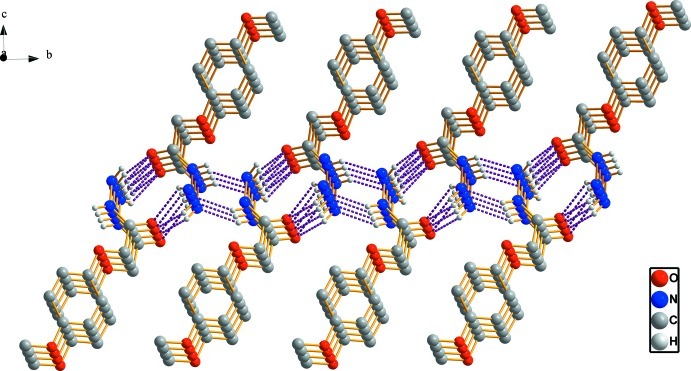
The structure of the infinite two-dimensional networks formed via hydrogen bonds (dashed lines). H atoms not involved in hydrogen bonds have been omitted for clarity.

## supplementary crystallographic information

### Comment

Hydrazides have been of great interest for many years because they have different biological activities and been used for the synthesis of various heterocyclic compounds (Khattab, 2005; Dutkiewicz *et al.*, 2009; Ozdemir *et al.*, 2009; Ashiq *et al.*, 2009; Zhang & Shi, 2009; Fun *et al.*, 2009, 2010*a*,*b*, 2011). In order to search for new hydrazide compounds with higher bioactivity, the title compound, was synthesized. Its molecular and crystal structures were determined. The molecular structure is shown in Fig. 1. In the crystal structure (Fig. 2), molecules are linked into infinite two-dimensional networks by the classical intermolecular N–H···N and N–H···O hydrogen bonds. For parameters of these interactions, see Table 1.

### Experimental

The title compound was synthesized by the reaction of 2-(4-methoxyphenoxy)acetate (1 mmol) with hydrazine hydrate 85% (1.1 mmol) in ethanol (15 ml) under reflux conditions (338 K) for 5 h. The solvent was removed and the solid product recrystallized from ethanol. After six days colourless crystals suitable for X-ray diffraction study were obtained.

### Refinement

The H atoms attached to N atoms were located in a difference Fourier map and allowed to refined freely. The remaining H atoms were placed in calculated positions (C–H = 0.93-0.97Å) and refined as riding atoms and with *U*_iso_(H) = 1.2 or 1.5*U*_eq_(C), respectively. The 609 Friedel pairs were measured.

### Crystal data

**Table:**

C_9_H_12_N_2_O_3_	*F*(000) = 416
*M**_r_* = 196.21	*D*_x_ = 1.400 Mg m^−^^3^
Orthorhombic, *P*2_1_2_1_2_1_	Mo *K*α radiation, λ = 0.71073 Å
Hall symbol: P 2ac 2ab	Cell parameters from 2044 reflections
*a* = 4.0964 (17) Å	θ = 2.3–25.2°
*b* = 6.382 (3) Å	µ = 0.11 mm^−^^1^
*c* = 35.608 (14) Å	*T* = 298 K
*V* = 930.9 (7) Å^3^	Block, colourless
*Z* = 4	0.30 × 0.25 × 0.18 mm

### Data collection

**Table:**

Bruker SMART APEXII CCD diffractometer	1631 independent reflections
Radiation source: fine-focus sealed tube	1503 reflections with *I* > 2σ(*I*)
Graphite monochromator	*R*_int_ = 0.021
φ and ω scans	θ_max_ = 25.1°, θ_min_ = 1.1°
Absorption correction: multi-scan (*SADABS*; Bruker, 2005)	*h* = −4→4
*T*_min_ = 0.969, *T*_max_ = 0.981	*k* = −7→7
4782 measured reflections	*l* = −42→37

### Refinement

**Table:**

Refinement on *F*^2^	Primary atom site location: structure-invariant direct methods
Least-squares matrix: full	Secondary atom site location: difference Fourier map
*R*[*F*^2^ > 2σ(*F*^2^)] = 0.030	Hydrogen site location: inferred from neighbouring sites
*wR*(*F*^2^) = 0.089	H atoms treated by a mixture of independent and constrained refinement
*S* = 0.88	*w* = 1/[σ^2^(*F*_o_^2^) + (0.070*P*)^2^ + 0.087*P*] where *P* = (*F*_o_^2^ + 2*F*_c_^2^)/3
1631 reflections	(Δ/σ)_max_ < 0.001
140 parameters	Δρ_max_ = 0.09 e Å^−^^3^
0 restraints	Δρ_min_ = −0.15 e Å^−^^3^

### Special details

**Table:**

Geometry. All s.u.'s (except the s.u. in the dihedral angle between two l.s. planes) are estimated using the full covariance matrix. The cell s.u.'s are taken into account individually in the estimation of s.u.'s in distances, angles and torsion angles; correlations between s.u.'s in cell parameters are only used when they are defined by crystal symmetry. An approximate (isotropic) treatment of cell s.u.'s is used for estimating s.u.'s involving l.s. planes.
Refinement. Refinement of *F*^2^ against ALL reflections. The weighted *R*-factor *wR* and goodness of fit *S* are based on *F*^2^, conventional *R*-factors *R* are based on *F*, with *F* set to zero for negative *F*^2^. The threshold expression of *F*^2^ > σ(*F*^2^) is used only for calculating *R*-factors(gt) *etc*. and is not relevant to the choice of reflections for refinement. *R*-factors based on *F*^2^ are statistically about twice as large as those based on *F*, and *R*-factors based on ALL data will be even larger.

### Fractional atomic coordinates and isotropic or equivalent isotropic displacement parameters (Å^2^)

**Table:**

	*x*	*y*	*z*	*U*_iso_*/*U*_eq_	
O1	0.7470 (4)	0.5028 (2)	0.28285 (3)	0.0575 (4)	
O3	−0.2287 (3)	1.24787 (16)	0.45169 (3)	0.0492 (3)	
O2	0.2900 (3)	0.86238 (18)	0.41321 (3)	0.0475 (3)	
N1	−0.0331 (4)	0.9456 (2)	0.47495 (3)	0.0375 (3)	
N2	−0.2052 (4)	0.9626 (2)	0.50921 (4)	0.0409 (4)	
C1	0.9213 (6)	0.3121 (3)	0.28446 (6)	0.0622 (6)	
H1A	1.1128	0.3301	0.2997	0.093*	
H1B	0.9844	0.2712	0.2596	0.093*	
H1C	0.7852	0.2055	0.2953	0.093*	
C3	0.4850 (5)	0.7769 (3)	0.31370 (4)	0.0445 (4)	
H3	0.4583	0.8404	0.2904	0.053*	
C7	0.6851 (5)	0.4962 (3)	0.35087 (5)	0.0456 (4)	
H7	0.7943	0.3690	0.3529	0.055*	
C2	0.6424 (4)	0.5884 (3)	0.31602 (4)	0.0418 (4)	
C6	0.5651 (4)	0.5936 (3)	0.38255 (4)	0.0434 (4)	
H6	0.5952	0.5317	0.4059	0.052*	
C9	−0.0652 (4)	1.0868 (2)	0.44833 (4)	0.0354 (4)	
C5	0.4022 (4)	0.7803 (2)	0.38004 (4)	0.0373 (4)	
C8	0.0994 (4)	1.0462 (2)	0.41147 (4)	0.0394 (4)	
H8A	−0.0641	1.0315	0.3919	0.047*	
H8B	0.2376	1.1643	0.4051	0.047*	
C4	0.3656 (4)	0.8742 (3)	0.34537 (4)	0.0432 (4)	
H4	0.2604	1.0029	0.3434	0.052*	
H1	0.085 (6)	0.833 (3)	0.4716 (6)	0.060 (6)*	
H2B	−0.073 (6)	1.041 (3)	0.5243 (5)	0.063 (6)*	
H2A	−0.384 (6)	1.036 (3)	0.5048 (5)	0.059 (6)*	

### Atomic displacement parameters (Å^2^)

**Table:**

	*U*^11^	*U*^22^	*U*^33^	*U*^12^	*U*^13^	*U*^23^
O1	0.0747 (9)	0.0574 (7)	0.0404 (7)	0.0081 (7)	0.0097 (7)	−0.0019 (5)
O3	0.0573 (8)	0.0379 (6)	0.0523 (7)	0.0129 (6)	0.0001 (6)	0.0029 (5)
O2	0.0616 (8)	0.0476 (7)	0.0333 (6)	0.0157 (7)	0.0007 (6)	0.0033 (5)
N1	0.0445 (8)	0.0316 (7)	0.0364 (7)	0.0048 (6)	−0.0010 (6)	0.0002 (5)
N2	0.0459 (9)	0.0384 (8)	0.0385 (7)	−0.0022 (7)	0.0027 (7)	−0.0012 (6)
C1	0.0675 (14)	0.0622 (12)	0.0570 (12)	0.0038 (11)	0.0112 (11)	−0.0109 (9)
C3	0.0533 (11)	0.0464 (9)	0.0339 (8)	−0.0017 (9)	−0.0018 (8)	0.0082 (7)
C7	0.0549 (11)	0.0383 (8)	0.0437 (9)	0.0064 (8)	0.0028 (8)	0.0054 (7)
C2	0.0445 (10)	0.0438 (9)	0.0369 (9)	−0.0057 (8)	0.0030 (7)	−0.0010 (7)
C6	0.0536 (10)	0.0429 (9)	0.0337 (8)	0.0032 (9)	−0.0010 (7)	0.0079 (7)
C9	0.0363 (8)	0.0298 (7)	0.0401 (8)	−0.0019 (7)	−0.0087 (7)	−0.0011 (7)
C5	0.0396 (9)	0.0370 (8)	0.0354 (8)	−0.0015 (7)	−0.0014 (7)	0.0010 (6)
C8	0.0430 (9)	0.0361 (8)	0.0390 (8)	0.0026 (7)	−0.0032 (7)	0.0037 (7)
C4	0.0495 (10)	0.0386 (8)	0.0416 (9)	0.0032 (8)	−0.0021 (7)	0.0069 (7)

### Geometric parameters (Å, º)

**Table:**

O1—C2	1.3701 (19)	C3—C2	1.367 (2)
O1—C1	1.412 (2)	C3—C4	1.377 (2)
O3—C9	1.2329 (18)	C3—H3	0.9300
O2—C5	1.3714 (19)	C7—C6	1.379 (2)
O2—C8	1.410 (2)	C7—C2	1.384 (2)
N1—C9	1.314 (2)	C7—H7	0.9300
N1—N2	1.413 (2)	C6—C5	1.368 (2)
N1—H1	0.88 (2)	C6—H6	0.9300
N2—H2B	0.91 (2)	C9—C8	1.498 (2)
N2—H2A	0.89 (2)	C5—C4	1.380 (2)
C1—H1A	0.9600	C8—H8A	0.9700
C1—H1B	0.9600	C8—H8B	0.9700
C1—H1C	0.9600	C4—H4	0.9300
			
C2—O1—C1	117.82 (13)	C3—C2—C7	119.18 (15)
C5—O2—C8	117.75 (11)	O1—C2—C7	124.33 (16)
C9—N1—N2	121.34 (14)	C5—C6—C7	120.86 (14)
C9—N1—H1	121.3 (13)	C5—C6—H6	119.6
N2—N1—H1	117.2 (14)	C7—C6—H6	119.6
N1—N2—H2B	104.8 (14)	O3—C9—N1	123.78 (15)
N1—N2—H2A	107.4 (13)	O3—C9—C8	118.28 (13)
H2B—N2—H2A	107.8 (19)	N1—C9—C8	117.92 (13)
O1—C1—H1A	109.5	C6—C5—O2	116.09 (13)
O1—C1—H1B	109.5	C6—C5—C4	119.31 (14)
H1A—C1—H1B	109.5	O2—C5—C4	124.59 (14)
O1—C1—H1C	109.5	O2—C8—C9	110.77 (12)
H1A—C1—H1C	109.5	O2—C8—H8A	109.5
H1B—C1—H1C	109.5	C9—C8—H8A	109.5
C2—C3—C4	120.96 (14)	O2—C8—H8B	109.5
C2—C3—H3	119.5	C9—C8—H8B	109.5
C4—C3—H3	119.5	H8A—C8—H8B	108.1
C6—C7—C2	119.79 (16)	C3—C4—C5	119.86 (15)
C6—C7—H7	120.1	C3—C4—H4	120.1
C2—C7—H7	120.1	C5—C4—H4	120.1
C3—C2—O1	116.49 (14)		
			
C4—C3—C2—O1	178.88 (17)	C7—C6—C5—C4	−1.7 (3)
C4—C3—C2—C7	−0.8 (3)	C8—O2—C5—C6	−175.24 (14)
C1—O1—C2—C3	177.62 (16)	C8—O2—C5—C4	5.8 (2)
C1—O1—C2—C7	−2.7 (3)	C5—O2—C8—C9	167.00 (14)
C6—C7—C2—C3	0.9 (3)	O3—C9—C8—O2	176.64 (14)
C6—C7—C2—O1	−178.80 (17)	N1—C9—C8—O2	−4.9 (2)
C2—C7—C6—C5	0.4 (3)	C2—C3—C4—C5	−0.5 (3)
N2—N1—C9—O3	4.3 (2)	C6—C5—C4—C3	1.7 (3)
N2—N1—C9—C8	−174.01 (14)	O2—C5—C4—C3	−179.35 (17)
C7—C6—C5—O2	179.31 (17)		

### Hydrogen-bond geometry (Å, º)

**Table:**

*D*—H···*A*	*D*—H	H···*A*	*D*···*A*	*D*—H···*A*
N2—H2*A*···O3^i^	0.89 (2)	2.51 (2)	3.155 (2)	130.4 (16)
N1—H1···N2^ii^	0.88 (2)	2.18 (2)	2.984 (2)	152.2 (18)
N2—H2*B*···O3^iii^	0.91 (2)	2.13 (2)	3.027 (2)	167.5 (18)

Symmetry codes: (i) *x*−1/2, −*y*+5/2, −*z*+1; (ii) *x*+1/2, −*y*+3/2, −*z*+1; (iii) *x*+1/2, −*y*+5/2, −*z*+1.
